# Metabolomic correlation-network modules in *Arabidopsis *based on a graph-clustering approach

**DOI:** 10.1186/1752-0509-5-1

**Published:** 2011-01-01

**Authors:** Atsushi Fukushima, Miyako Kusano, Henning Redestig, Masanori Arita, Kazuki Saito

**Affiliations:** 1 RIKEN Plant Science Center, Kanagawa 230-0045, Japan; 2 Kihara Institute for Biological Research, Yokohama City University, Kanagawa 244-0813, Japan; 3 The University of Tokyo, Tokyo 113-0033, Japan; 4 Keio University, Yamagata 997-0052, Japan; 5 Chiba University, Chiba 263-8522, Japan

## Abstract

**Background:**

Deciphering the metabolome is essential for a better understanding of the cellular metabolism as a system. Typical metabolomics data show a few but significant correlations among metabolite levels when data sampling is repeated across individuals grown under strictly controlled conditions. Although several studies have assessed topologies in metabolomic correlation networks, it remains unclear whether highly connected metabolites in these networks have specific functions in known tissue- and/or genotype-dependent biochemical pathways.

**Results:**

In our study of metabolite profiles we subjected root tissues to gas chromatography-time-of-flight/mass spectrometry (GC-TOF/MS) and used published information on the aerial parts of 3 *Arabidopsis *genotypes, Col-0 wild-type, *methionine over-accumulation 1 *(*mto1*), and *transparent testa4 *(*tt4*) to compare systematically the metabolomic correlations in samples of roots and aerial parts. We then applied graph clustering to the constructed correlation networks to extract densely connected metabolites and evaluated the clusters by biochemical-pathway enrichment analysis. We found that the number of significant correlations varied by tissue and genotype and that the obtained clusters were significantly enriched for metabolites included in biochemical pathways.

**Conclusions:**

We demonstrate that the graph-clustering approach identifies tissue- and/or genotype-dependent metabolomic clusters related to the biochemical pathway. Metabolomic correlations complement information about changes in mean metabolite levels and may help to elucidate the organization of metabolically functional modules.

## Background

Combining and integrating different 'omics' data such as transcript-, protein-, and metabolite levels and enzyme activities is essential for a full understanding of the nature of the cellular metabolism as a system [1-4]. With respect to transcript levels, a large amount of microarray data is publicly available for *Arabidopsis thaliana*, a model plant. Such large datasets facilitate the construction of gene co-expression databases [5] and the survey of transcriptome organization [6-8]. Integrating transcript- and metabolite data by, for example, studying the correlation relationships among profiled data, facilitates the characterization of unknown gene functions, and furthers our understanding of plant cellular systems [9-11].

The correlation between variables (e.g. genes and metabolites) is also important for multivariate statistical analyses such as principal component analysis (PCA) and hierarchical cluster analysis. Typical metabolite-profiling data show a few, but significant correlations among metabolite levels when data sampling is repeated across individuals grown under strictly controlled conditions [12]. The metabolomic correlation as well as gene co-expression are not always in agreement with known biochemical pathways. Metabolomic correlation approaches have highlighted some properties (e.g. modularity and scale-freeness) in several species including plants [13-16]. Steuer et al. [17], who provided a relationship between the structure of a metabolomic-correlation network and a metabolic reaction network using a Jacobian matrix, found that the relationship is not simple. They pointed out that small fluctuations such as glucose availability can result in a certain correlation pattern and persist through metabolic pathways. Using metabolic control analysis (MCA) and correlation analysis based on metabolomic data, Camacho et al. [18] suggested that metabolites are strongly correlated when they respond in the same directions to all perturbations (fluctuations) in enzyme levels. For example, mass conservation and chemical equilibrium were suggested as one origin of a high correlation. Muller-Linow et al. [19] applied network similarity, a graph-theoretic parameter, to compare metabolomic correlation networks with biochemical reactions derived from the KEGG database [20]. They reported that these networks were in disagreement and that closeness in metabolomic correlation is not an indicator of closeness in biochemical networks. Studies on the effect of changes in environmental conditions and temporal- and spatial assessments of the topology of metabolomic correlation networks have been reported [19,21,22]. Further investigation of the properties of metabolomic correlation networks may discover whether highly connected metabolites, the so-called 'modules', in the correlation network reflect known biochemical pathways.

We investigated similarities and dissimilarities in metabolomic correlations in the aerial parts of 3 *Arabidopsis *genotypes, Col-0 wild-type (WT), *methionine-over accumulation 1 *(*mto1*) [23], and *transparent testa4 *(*tt4*) [24]. Elsewhere [25] we reported that the mutation in cystathionine γ-synthase (CGS) and/or the over-accumulation of methionine (Met) strongly affect the correlation networks in aerial parts of *mto1*. In the present study, using gas chromatography-time-of-flight/mass spectrometry (GC-TOF/MS), we measured the relative metabolite levels in root samples of the 3 *Arabidopsis *genotypes to assess tissue- and/or genotype-dependent changes in their metabolite levels. We systematically compared the metabolomic correlations observed in 2 different datasets, the roots and the aerial parts. Multivariate statistical analyses showed the distinct metabolome of these plants and tissues. We then constructed correlation networks by pair-wise correlation between the metabolites and performed graph clustering using the DPClus algorithm [26] that efficiently extracts densely connected metabolites in a large-scale network. We then evaluated the obtained clusters with KEGG [20] enrichment analysis. Our results demonstrate that changes in each network topology are tissue- and/or genotype-dependent and that they reflect, at least partially, known biochemical pathways in *Arabidopsis*.

## Results

### Metabolic phenotypes of the roots of 3 *Arabidopsis *genotypes

**Figure 1:**
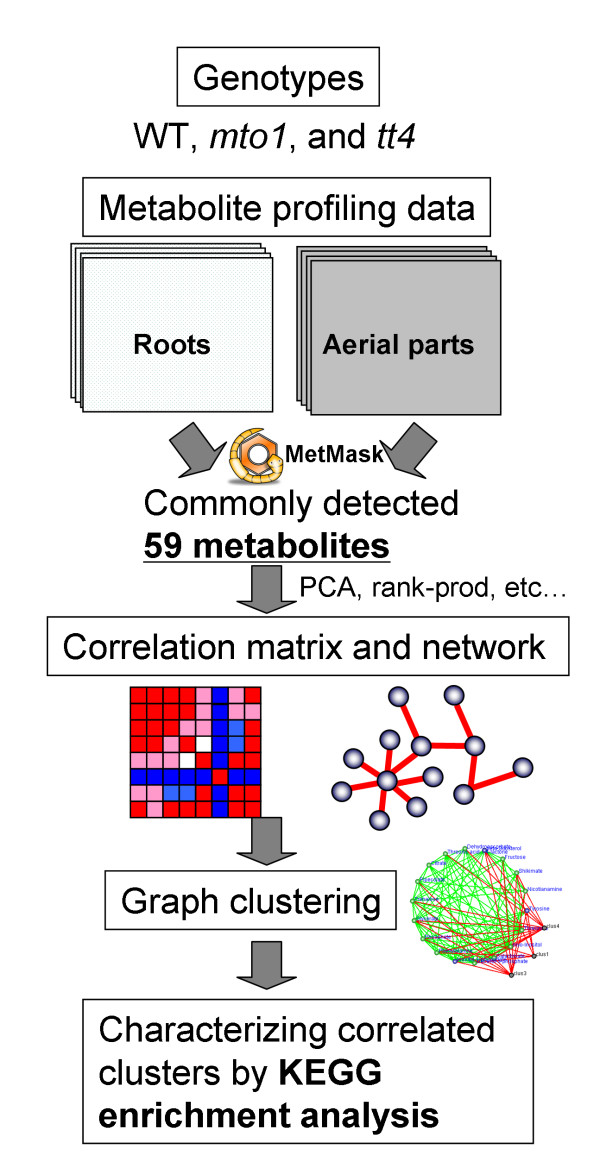
**Outline of the procedure for comparing tissue- and/or genotype-dependent metabolomic correlations in *Arabidopsis***. Three genotypes [*methionine-over accumulation 1 *(*mto1*), *transparent testa4 *(*tt4*), and wild-type (WT)] were studied. Samples from aerial parts and roots were analyzed by GC-TOF/MS-based metabolite profiling and changes in mean metabolite levels were assessed and calculated by multivariate statistical analysis PCA, the rank product method, and the metabolomic correlations by the Spearman correlation coefficient. Then we constructed the metabolomic correlation networks. Using DPClus [26] we extracted densely connected metabolites. Lastly, we characterized each cluster based on the KEGG enrichment analysis to assess statistical significance.

The experimental workflow is shown in Figure 1. Roots of Col-0 wild-type (WT), *mto1*, and *tt4 *mutants were sampled and analyzed. We detected 166 metabolite peaks including mass spectral tags (MSTs) [27] by the GC-TOF/MS-based metabolite profiling we established for *Arabidopsis *[25]. Of these, 83 were known metabolites including carbohydrates, amino-, fatty-, and organic acids and vitamins, and others were secondary metabolites (Additional file 1). For comparisons with data from aerial parts we selected 59 commonly-detected metabolites in both datasets using MetMask http://metmask.sourceforge.net[28], a tool for metabolite identifier linking.

**Figure 2:**
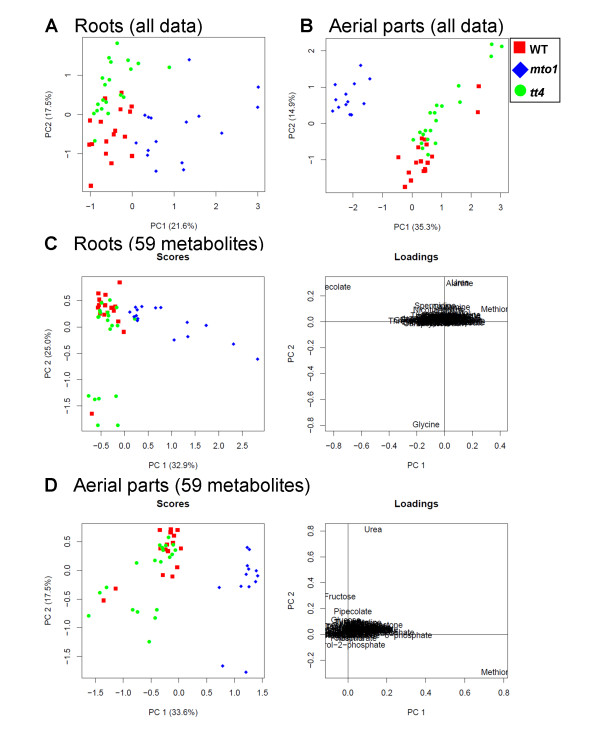
**Principal component analyses of the roots and aerial parts of 3 *Arabidopsis *genotypes**. Score scatter plots for roots (A) and aerial parts (B) using all detected peaks are shown. Score and loading plots using 59 commonly detected metabolites for roots (C) and aerial parts (D) are presented. These data include 53 root samples (WT, *n *= 17; *mto1*, *n *= 16; and *tt4*, *n *= 20) and 50 samples from aerial parts (WT, *n *= 17; *mto1*, *n *= 13; and *tt4*, *n *= 20). Symbols: red squares, wild-type (WT); blue diamonds, *mto1*; green circles, *tt4*. Data on the aerial parts were from the work of [25].

By PCA we visually inspected the metabolite profiles of the roots and aerial parts based on commonly detected metabolites and all peaks (Figure 2). To confirm the most important principal components (PC) of these samples we prepared score plots for each dataset. As in aerial parts, samples of *mto1 *roots were clearly separated while samples of *tt4 *roots were close to WT along the second component axis (Figures 2A and 2B). The loading plots highlighted and visualized metabolites with a significant role in genotype separation. For example, methionine (Met), urea, glycine, and pipecolate had a strong impact on the roots and aerial parts of *mto1 *and *tt4 *(primarily the first principal component). The complete list of metabolites that contributed to the separation of the mutant profile groups from WT (discriminative metabolites) is shown in Additional file 2. In the PCA score plots of the 59 metabolites, the first principal component (PC1) clearly discriminated between *mto1*- and the control samples (Figures 2C and 2D). To obtain further insights into the interpretation of our PCA results, we re-calculated PCA by removing the four most influencing metabolites (methionine, urea, glycine, and pipecolate) from the original data matrices. Additional file 3 shows the score and loading plots of roots (A) and aerial parts (B) using 55 metabolite datasets. The PCA plots revealed that asparagine and glutamine strongly contributed to PC1 separation in both the roots and aerial parts (see Discussion).

### The number of significant correlations depends on the tissue/genotype and their differential correlations

**Figure 3:**
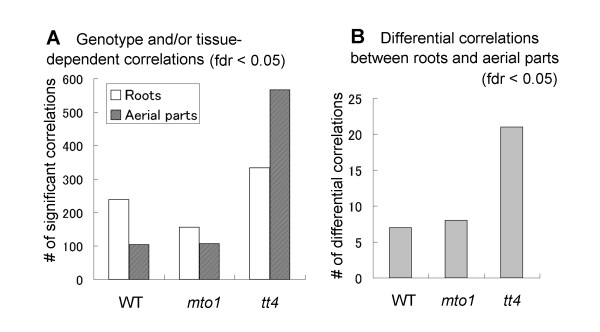
**Statistics of correlation analyses among the 3 *Arabidopsis *genotypes**. (A) Genotype- and/or tissue-dependent correlations and (B) differential correlations between roots and aerial parts are shown. A significance level set at fdr < 0.05, local false discovery rate. fdr was calculated using fdrtool package [29]. The resulting correlations are all listed in Additional file 4.

To evaluate the correlations among the metabolite profiles observed in each tissue and genotype we calculated the Spearman correlation coefficient (*r*). The significant correlations (local false discovery rate (fdr) < 0.05; see ref [29]) for each tissue and each genotype are shown in Figure 3. All resultant correlations are listed in Additional file 4.

We first examined the number of significant correlations between tissues. We found that the number of significant correlations in WT and *mto1 *roots was larger than in their aerial parts (Figure 3A); it was also much larger in the aerial parts of *tt4 *than its roots. Then, we compared differences between correlations in roots and aerial parts using Fisher Z-transformation (see Methods). Similarly, we also compared differential correlations between genotypes. The number of statistical differences between correlations in roots and aerial parts in WT, *mto1*, and *tt4 *was 7, 8, and 21, although there were no significant changes in the correlations between genotypes except for 6 differential correlations between WT and *tt4 *in roots (fdr < 0.05) (Figure 3B and Additional file 4). Our findings imply that in both tissue-types, the *mto1 *mutation strongly affects the metabolomic correlation network, and that there are many correlations intensified by a lack of the chalcone synthase (CHS) gene. Our observations also suggest that the tendency for changes in the number of significant correlations is similar within the studied tissues of the 3 genotypes.

### Construction of a metabolomic correlation network and selection of the correlation threshold

For visualization and to gain insights into the metabolomic correlations for each tissue and genotype we constructed correlation networks. The selection of an appropriate threshold is important for the construction of such a correlation network, if it is too high the generated network will be sparse, if too low, only a large connected component will be produced. To assess threshold-dependent changes in the network topology we computed 6 graph-theoretic measures (Additional files 5 and 6), i.e., the graph density, clustering coefficient, average degree, average path length, number of connected components, and the number of edges (for example, see [30]). We also compared the statistics of the real data and 100 randomized data (dots in Additional files 5 and 6). We observed that the number of connected components showed a transition from small to large at a correlation threshold of 0.5 (arrows in Additional file 5). Therefore, we set the threshold at *r *≥ 0.5. Although a threshold does not guarantee explicit biological significance, we found that examining various statistics among the constructed networks was useful for selection.

### Graph clustering identifies modules significantly enriched for metabolites contained in biochemical pathways

**Figure 4:**
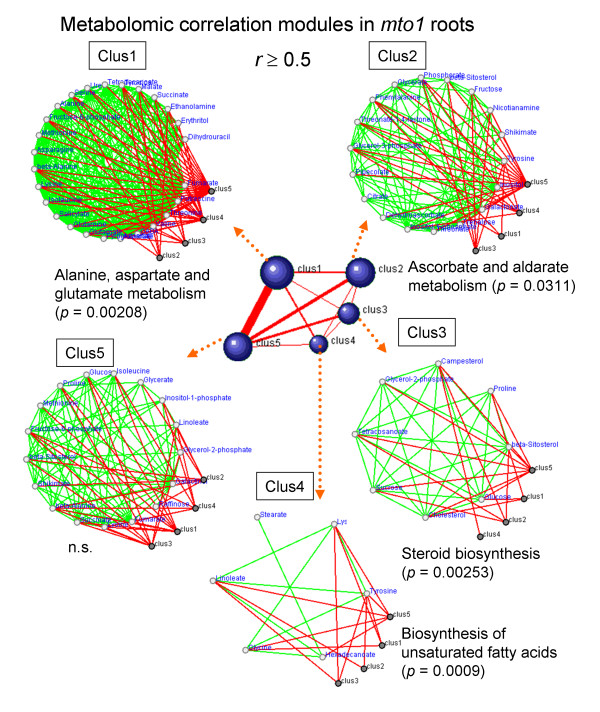
**Graph clustering of metabolomic correlated modules in *mto1 *roots (threshold *r *≥ 0**.5). Using the DPClus algorithm [26] we extracted 5 clusters in *mto1 *roots. The significant metabolic pathways were assigned by KEGG enrichment analysis (see Methods). The central graph consisting of 5 blue clusters and 10 red edges was extracted by DPClus. Each blue cluster contains densely connected metabolites (see Clus1 to 5). Small white nodes in the clusters indicate metabolites. Clus1 (top, left) shows the first cluster of the hierarchical graph. The internal nodes of the clusters are connected by green edges; neighboring clusters are connected by red edges. Both *c_p _*and density values, DPClus parameters, were set to 0.5. We used overlapping-mode in DPClus settings. The results obtained in the other 2 genotypes are shown in Additional file 8.

**Figure 5:**
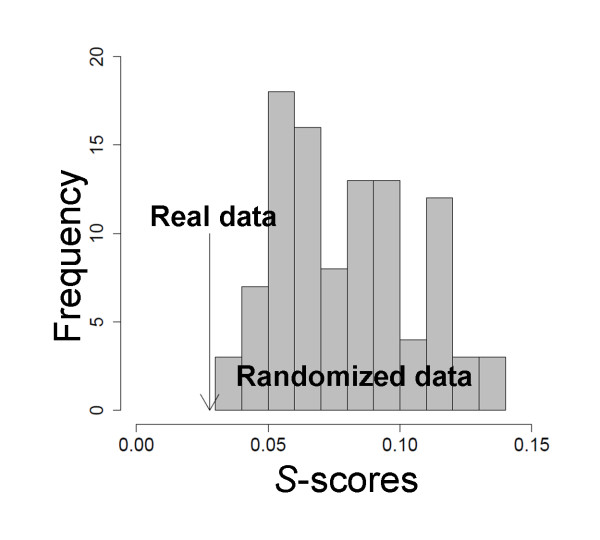
**Assessment of statistical significance in the resulting metabolomic clusters in *mto1 *roots using DPClus**. The best *p*-values for the KEGG pathways, averaged over all clusters (*S*-score; see Methods), were compared with the corresponding values of 100 randomly generated clusters (grey histogram). The averaged *S*-score (black solid arrow) was 0.0278, indicating that real- scored significantly better than randomized data.

At *r *≥ 0.5, DPClus [26] identified 5, 5, and 4 clusters in the metabolomic correlation network for the root samples of WT, *mto1*, and *tt4*, respectively; they ranged in size from 6 to 29 metabolites. For the aerial parts of WT, *mto1*, and *tt4 *we obtained 5, 7, and 5 clusters, respectively; they ranged in size from 4 to 34 metabolites (Additional file 7). We posit that this difference in the number of clusters reflects differences in the network topology, which, in turn, is tissue- and genotype-dependent. To assess the significance of the clusters we used the over-represented KEGG pathways (so-called KEGG enrichment analysis) in the obtained- and randomized clusters (see Methods and Table 1). The results of graph clustering with KEGG enrichment analysis for *mto1 *roots are presented in Figure 4. Our findings on the other genotypes are listed in Table 2; they are visualized in Additional file 8. The clusters obtained with the graph clustering method involved the enriched KEGG pathway and included the 'alanine-, aspartate-, and glutamate metabolism' (*p *= 0.0021) in *mto1 *roots (Figure 4). The statistical relevance of this approach was evaluated with the *S*-value [6] that can be used for assessing the significance of the clustering results based on KEGG pathways (see Methods). As shown in Figure 5, the averaged best *p*-value for significantly-enriched KEGG pathways was better in real- than randomized data.

**Table 1:** Two-by-two contingency table

	within DPClus cluster	without DPClus cluster	total
KEGG pathway	**a**	**b**	**a**+**b**

non-KEGG pathway	**c**	**d**	**c**+**d**

totals	**a**+**c**	**b**+**d**	**n**

**a **is the number of metabolites in the KEGG pathway belonging to the DPClus cluster, **a**+**b **indicates the total number of metabolites in the KEGG pathway, **a**+**c **the number of metabolites in the DPClus cluster, and **n **the total number of metabolites in the profiling data. Rows reflect presence/absence within the KEGG pathway. Columns contain each of the 2 DPClus clusters. For each KEGG pathway the metabolites belonging to the 2 comparison groups are shown.

**Table 2:** List of enriched KEGG pathways in each cluster detected by DPClus

	*p*-value_min_	Metabolism
WT, roots		
Clus1	0.0002	Aminoacyl-tRNA biosynthesis
Clus2	n.s.	-
Clus3	0.0061	Steroid biosynthesis
Clus4	0.0186	Galactose metabolism
Clus5	0.0041	Carbon fixation in photosynthetic organisms
		
*mto1*, roots		
Clu1	0.0021	Alanine, aspartate and glutamate metabolism
Clu2	0.0311	Ascorbate and aldarate metabolism
Clu3	0.0025	Steroid biosynthesis
Clu4	0.0009	Biosynthesis of unsaturated fatty acids
Clu5	n.s.	-
		
*tt4*, roots		
Clus1	0.0224	Biosynthesis of alkaloids derived from shikimate pathway
Clus2	0.0102	Steroid biosynthesis
Clus3	0.0012	Nitrogen metabolism
Clus4	0.0139	Purine metabolism
		
WT, aerial parts		
Clus1	0.0017	Galactose metabolism
Clus2	0.0422	Biosynthesis of plant hormones
Clus3	0.0041	Glucosinolate biosynthesis
Clus4	0.0220	Glycerophospholipid metabolism
Clus5	0.0005	Biosynthesis of unsaturated fatty acids
		
*mto1*, aerial parts		
Clus1	0.0205	Alanine, aspartate and glutamate metabolism
Clus2	0.0019	Galactose metabolism
Clus3	n.s.	-
Clus4	0.0146	Tropane, piperidine and pyridine alkaloid biosynthesis
Clus5	n.s.	-
Clus6	n.s.	-
Clus7	n.s.	-
		
*tt4*, aerial parts		
Clus1	0.0197	Aminoacyl-tRNA biosynthesis
Clus2	0.0038	Steroid biosynthesis
Clus3	0.0000	Galactose metabolism
Clus4	0.0045	Biosynthesis of unsaturated fatty acids
Clus5	n.s.	-

### Predominant pathways in clusters reflect tissue- and/or genotype-dependent metabolomic regulation

Except for the clusters found in the aerial parts of WT and *mto1*, the detected clusters tended to include steroid biosynthetic pathways (Table 2). The strong correlation between sterols confirmed our earlier observations for aerial parts [25]. Metabolites belonging to fatty-acid biosynthesis tended to cluster together. The clusters we commonly found in the aerial parts of the 3 genotypes were galactose metabolism. The largest cluster for the aerial parts and roots of *mto1 *were 'alanine, aspartate, and glutamate metabolism.' This suggests that the mutation resulting in Met over-accumulation can affect the metabolic pathway of the aspartate family. Metabolite enrichment analysis using other *mto *mutants also extracted the same pathways [31]. The largest cluster in the roots of *tt4 *was 'biosynthesis of alkaloids derived from the shikimate pathway.' For both tissues, *tt4*, which lacks the gene encoding CHS [24], showed less pronounced changes in the metabolite levels belonging to this biosynthetic pathway (see Discussion and Additional file 2).

## Discussion

We focused on the changes in the topology of metabolomic correlation networks of 3 *Arabidopsis *genotypes (WT, *mto1*, and *tt4*). When comparing the WT, the increases and decreases in the number of significant correlations for *mto1 *and *tt4 *were similar in their aerial parts and roots. Our comparative studies showed strongly altered metabolomic correlations in the roots of *mto1*. As did Kusano et al. [25] in aerial parts, we observed a marked increase in the number of significant correlations in the roots of *tt4 *compared to the roots of WT. As our study imposed no environmental perturbations on the 3 genotypes, we suggest that the observed changes in the topology of metabolomic correlation networks are attributable to underlying tissue- and/or genotype-dependent biochemical regulations.

Graph clustering using DPClus [26] yielded densely connected metabolites on the metabolomic correlation networks. KEGG enrichment analysis to assess the statistical significance of the detected clusters demonstrated specific differences in the clusters in the enriched pathways. We posit that the assigned KEGG pathways for each cluster reflect differences in underlying genetic properties and in tissue- and/or genotype-dependent biochemical regulations.

The largest cluster in *mto1 *roots was assigned as the'alanine, aspartate, and glutamate metabolism'. Since *mto1 *mutants are characterized by mutations involving the aspartate pathway, the approach based on graph-clustering proved itself useful for characterizing genotypes. Although there were no significant enriched KEGG pathways in cluster 5 in Figure 4, this cluster may represent the extensive coordination among biosynthetic pathways involved in glycolysis, the tricarboxylic acid (TCA)-cycle, and stress-responsive metabolites in *mto1 *roots. Our approach may yield new insights into the organization of metabolites in the functional pathways of a given organism. In *tt4 *roots, the largest cluster was 'biosynthesis of alkaloids derived from the shikimate pathway'. This cluster contained metabolites associated with the biosynthetic pathways of the TCA cycle and with glycolysis because this KEGG pathway covered extensive pathways from glycolysis to alkaloids via shikimate for the biosynthesis of aromatic amino acids including phenylalanine, tyrosine, and tryptophan. The mutation in the CHS gene appears not to affect the biosynthesis of alkaloids in *Arabidopsis*. We posit that the model plant *Arabidopsis *lacks the production of complex alkaloids, although homologues for many enzymes associated with alkaloid biosynthesis have been detected in its genome [32]. Our multivariate statistical analysis suggests that the mutation of CHS may influence nitrogen assimilation (Additional file 3). Furthermore, the third cluster (Clus3) in *tt4 *roots was significantly enriched for metabolites contained in 'nitrogen metabolism' (Table 2), suggesting that our approach may reflect underlying changes in the metabolism by lacking of CHS in *Arabidopsis*. Taken together, our observations demonstrate that variations in the topology of correlation networks are tissue and/or genotype-dependent and reflect at least partially known biochemical pathways in *Arabidopsis*.

Earlier studies indicated that the interpretation of metabolomic correlations requires careful evaluation [12,17,18]. Our work demonstrates that graph clustering can be used to gather metabolites belonging to the metabolic pathways that change in response to different regulations that in turn are dependent on the tissue and/or genotype. Our analyses differ from those of [19] in that we examined the densely connected metabolites in the correlation network by statistical KEGG enrichment analysis instead of using network similarity or proximity. Graph clustering approaches have been effectively applied to gene co-expression networks for extracting functional, densely connected genes [6,33-35]. We showed that the approach is also effective for metabolomic correlations.

Although we only evaluated 3 genotypes, the approach based on graph clustering seems to be useful and applicable to the monitoring of changes in the topology of correlation networks. As in the gene co-expression analysis that involves thousands of microarrays, there were few negative correlations in this study. For instance, the number of significant negative correlations (*r *< 0 and fdr < 0.05) in the roots and aerial parts of WT was 17 and 6, respectively. Therefore, we deliberately did not use negative correlations. Other similarity measures such as mutual information [36] and partial correlation [37], instead of Spearman correlation coefficients, may be applied in this approach. Further studies are necessary to assess to what extent these measures affect the graph clustering results of metabolomic correlation networks.

There are few systematic comparisons of metabolomic correlations among time series data [19]. Temporal and spatial assessment of the topology in the metabolomic correlation network may contribute to the development of novel data-mining methods and to the discovery of biomarkers [38]. There are also few published systematic metabolomic comparisons under stress conditions. Szymanski et al. [21] demonstrated that the correlation network approach can help to identify stress-dependent regulatory mechanisms in *Escherichia coli*. Our current report is a logical extension of our continued effort to understand the regulation of the primary (especially Met biosynthesis [25]) and the secondary (especially flavonoid biosynthesis [39]) metabolism in *Arabidopsis*. Our approach contributes to the generation of new testable hypotheses for further experiments and may expand our fundamental understanding of the metabolic behaviors affected by genetic and/or environmental perturbations. Such information will contribute to the characterization of unknown gene function(s) and help in the high-throughput screening of metabolic phenotypes ranging from experimental models to crops [40] and disease studies [41].

## Conclusions

This study demonstrated that (1) differences in mutations and/or tissues affect changes in the topology of metabolomic correlation networks; (2) graph clustering yields tissue- and/or genotype-dependent metabolomic clusters related to respective biochemical pathways; and (3) metabolomic correlations complement information on changes in mean metabolite levels and the approach based on metabolomic correlations yields insights into the organization and regulation of metabolic modules.

## Methods

### Plant materials, growth, and harvest

For metabolomic correlation analysis we sampled tissues from the roots of *Arabidopsis thaliana *Col-0 wild-type (WT), *methionine over-accumulation 1 *(*mto1*) [23], and *transparent testa4 *(*tt4*) [24]. Data on the aerial part were from [25]. The number of biological replicates in roots was 53 (17 × WT, 16 × *mto1*, and 20 × *tt4*). We pooled 3 root samples as a batch for metabolite profiling.

### Metabolite profiling

As described in [25], each sample was extracted, derivatized, and analyzed by GC-TOF/MS. Briefly, we pre-processed all raw data using custom MATLAB software (version 6.5; Mathworks, Natick, MA, USA) for hyphenated data analysis (HDA) [42]; it performs baseline correction, peak alignment, and peak deconvolution. For metabolite identification we used the Golm Metabolome Database (GMD) [27,43] and our in-house mass spectral libraries.

### Statistical data analysis

Metabolomic data were log2 transformed and then statistically analyzed using the rank product method [44] to identify differentially changed metabolites with the Bioconductor 'RankProd' package. Significantly changed metabolites showed a false discovery rate (FDR) < 0.05. The FDR value in the rank product was obtained with 1,000 random permutations. PCA was performed with the Bioconductor 'pcaMethods' package [45].

To reduce outlier-related artifacts, the Spearman rank correlation (*r*), which is relatively robust to outliers and is based only on monotonicity, was used for comparative metabolomic correlations. The statistical significance of the correlation coefficient (*r*) was tested against the hypothesis of no correlation (*r *= 0) according to *t*-statistics: , where *n *shows the sample size.

To identify differences between correlations, we tested whether two correlations manifested different strengths. Using Fisher Z-transformation, two correlation coefficients were transformed

where *r*_1 _and *r*_2 _are the correlation coefficient for each of the two tissues (or two genotypes), and *n*_1 _and *n*_2 _indicate the number of replicates for each of the two tissues (or genotypes) for each metabolite-metabolite pair. The local false-discovery rate (fdr) was determined with the 'fdrtool' package [29]. The significance level was set at fdr < 0.05. All statistical tests were performed with the R program http://www.r-project.org/.

### Network analyses and graph clustering

To identify co-accumulated metabolite groups we used DPClus [26], a graph clustering algorithm that can extract densely connected nodes as a cluster. It is based on density- and periphery tracking of clusters. DPClus is freely available from http://kanaya.naist.jp/DPClus/. In this study, we used the overlapping-mode with the DPClus settings because we are confident that extracting clusters with the overlapping-mode is consistent with the overlapping of many of the metabolic pathways and protein complexes. We set the parameter settings of cluster property *c_p_*; density values were set to 0.5. The resulting clusters are listed in Additional file 7. All network statistics such as graph density were calculated in R with the 'igraph' package [46].

### Generation of randomized clusters and assessment of clustered modules in metabolomic correlation networks

To assess the statistical significance of clusters obtained by graph clustering, we subjected datasets of the aerial parts and roots of the 3 *Arabidopsis *genotypes and 100 randomly-generated sets of clusters to KEGG enrichment. Each randomized cluster was created by permutation of the metabolite names without changing the cluster size. The assessment was done using the *S*-value [6]: , where *n *shows the number of clusters and *i *a cluster. This value is based on the best *p*-value, *p_min_*, for KEGG pathway enrichment in each cluster. The best *p*-values were averaged over all clusters to provide the *S*-value.

The significance of the KEGG pathway in clusters is represented by the *p*-value, which shows Fisher's exact probabilities based on two-by-two contingency tables: Table 1 is a 2 × 2 table ("KEGG pathway", "non-KEGG pathway", "within DPClus cluster", "without DPClus cluster") used by Fisher's exact test, where significance is defined as a *p*-value (obtained from the test for DPClus clusters) that is less than or equal to a pre-set level of significance (*p *< 0.05). The probability of obtaining any set of values was given by the hypergeometric distribution. A full list of the KEGG pathways used in this study is shown in Additional file 9.

## Authors' contributions

AF designed this research, analyzed the data, and wrote the manuscript. MK analyzed the metabolite profiling and edited the manuscript; HR assisted in chemical identifier linking and edited the manuscript; AF, MK, and MA interpreted the data; MA and KS supervised the project and edited the manuscript. All authors read and approved the final manuscript.

## Supplementary Material

Additional file 1 **Raw data matrix used in this study**. The normalized data matrix of 166 peak areas of extracted mass spectra for 53 root samples (17 × WT, 16 × *mto1*, and 20 × *tt4*; see also Methods). Click here for file

Additional file 2 **Discriminative metabolites of PCA loadings and significant changes in the metabolite levels of WT, *mto1*, and *tt4***. All 59 metabolites that contributed to the separation of the mutant profiles group from WT are shown: *mto1*/WT in the roots (A) and aerial parts (B) and *tt4*/WT in the roots (C) and aerial parts (D). Differentially changed metabolites were identified with the rank product method (see Methods). Significantly changed metabolites manifested a false discovery rate (FDR) < 0.05. Click here for file

Additional file 3 **PCA by removing four metabolites (methionine, urea, glycine, and pipecolate) in (A) roots and (B) aerial parts using datasets including 55 metabolites**. See also the legend to Figure 2. Click here for file

Additional file 4 **List of metabolomic correlations based on Spearman's coefficient for the aerial parts and roots of WT, *mto1*, and *tt4***. *r*_1 _and *r*_2 _represent the coefficient of WT and the mutant, respectively. *p*_1 _and *p*_2 _are the *p*-value of the correlation test for WT and the mutant, respectively. '*p *(diff)' is the *p*-value of comparing correlations using Fisher Z-transformation. (*r*_1 _- *r*_2_) is the subtraction of *r*_2 _from *r*_1_. 'fdr' is the local false discovery rate (fdr)-controlled *p*-value using the 'fdrtool' package [29]. Click here for file

Additional file 5 **Correlation network properties of the 59 metabolites in the aerial parts across a range of correlation coefficients**. Networks were constructed for a range of correlation thresholds from 0 to 1.0 by 0.01 increments, and each resulting network was calculated for: (A) the graph density - the ratio of the number of edges and the number of possible edges, (B) the clustering coefficient, (C) the average degree of all nodes, (D) the average path length, (E) the number of connected components, and (F) the number of metabolite-metabolite correlations (edges) in the network. Within each plot, black solid circles represent the observed data points; black dots represent 100 randomized data. This calculation was performed to generate a null distribution. Click here for file

Additional file 6 **Correlation network properties of the 59 metabolites in roots across a range of correlation coefficients**. See details in the legend for Additional file 5. Click here for file

Additional file 7 **Resulting clusters detected by the DPClus algorithm**. For details, see the manual of DPClus http://kanaya.naist.jp/DPClus/. Click here for file

Additional file 8 **Visualization of all DPClus clusters**. See details in the legend for Figure 4. Click here for file

Additional file 9 **List of KEGG pathways used in this study**. Click here for file
