# Supplementary figures and images for: Metabolomic correlation-network modules in Arabidopsis based on a graph-clustering approach
Source: BMC Syst Biol. 2011 Jan 1;5:1. doi: 10.1186/1752-0509-5-1 (PMC3030539; doi:10.1186/1752-0509-5-1)

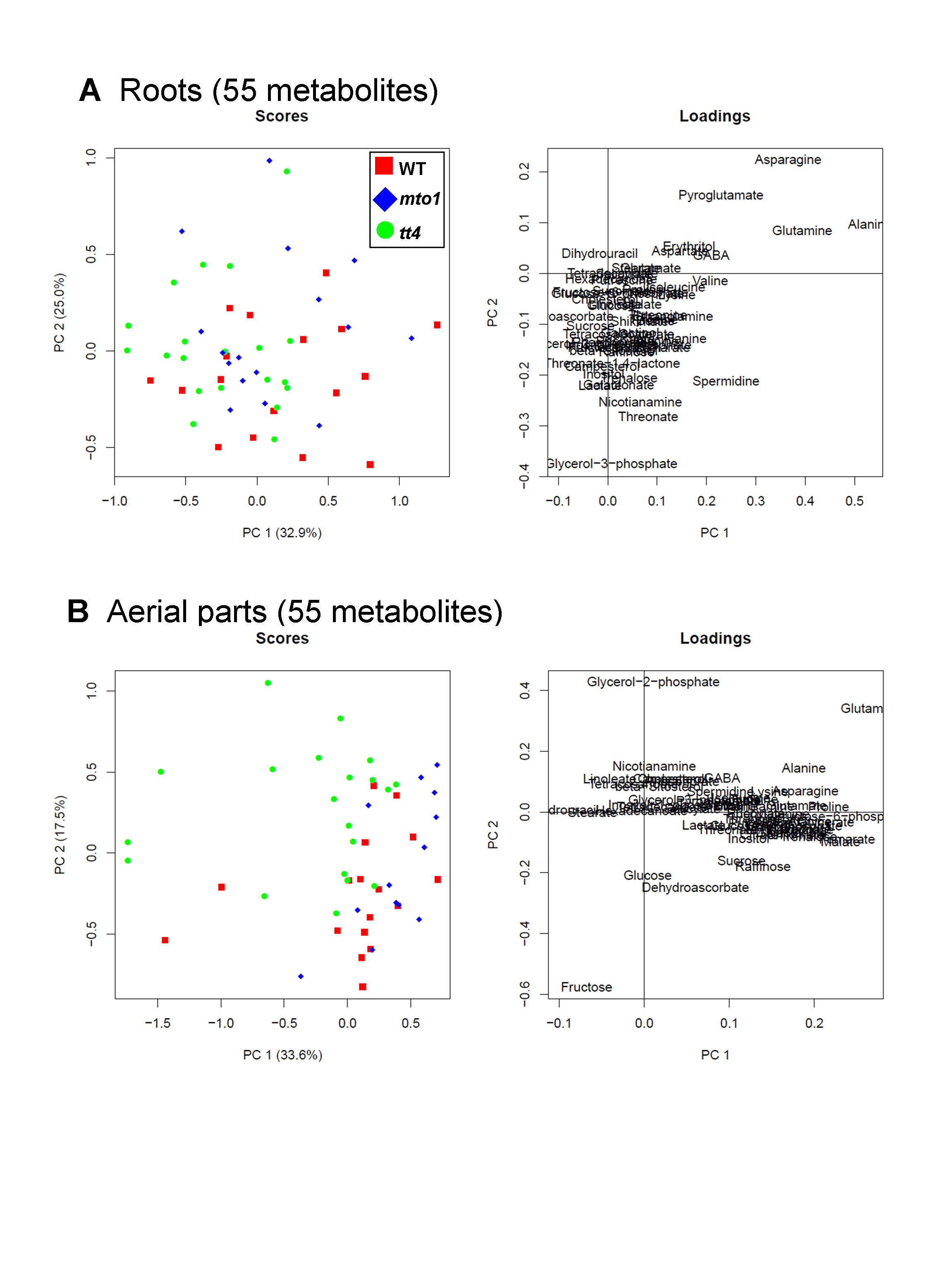

Supplement: Additional file 3 — PCA by removing four metabolites (methionine, urea, glycine, and pipecolate) in (A) roots and (B) aerial parts using datasets including 55 metabolites. See also the legend to Figure 2. [file 1752-0509-5-1-S3.TIFF]

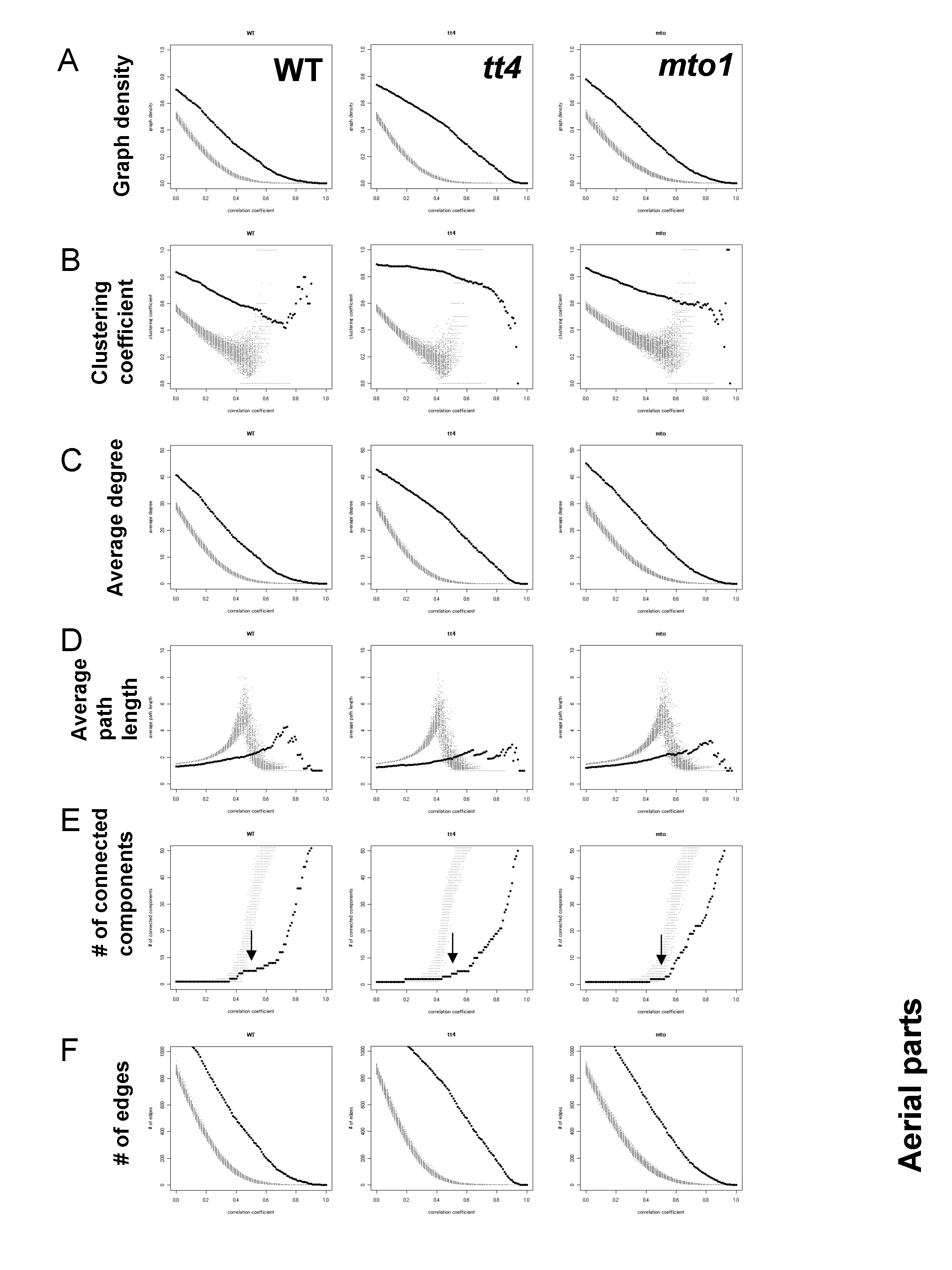

Supplement: Additional file 5 — Correlation network properties of the 59 metabolites in the aerial parts across a range of correlation coefficients. Networks were constructed for a range of correlation thresholds from 0 to 1.0 by 0.01 increments, and each resulting network was calculated for: (A) the graph density - the ratio of the number of edges and the number of possible edges, (B) the clustering coefficient, (C) the average degree of all nodes, (D) the average path length, (E) the number of connected components, and (F) the number of metabolite-metabolite correlations (edges) in the network. Within each plot, black solid circles represent the observed data points; black dots represent 100 randomized data. This calculation was performed to generate a null distribution. [file 1752-0509-5-1-S5.TIFF]

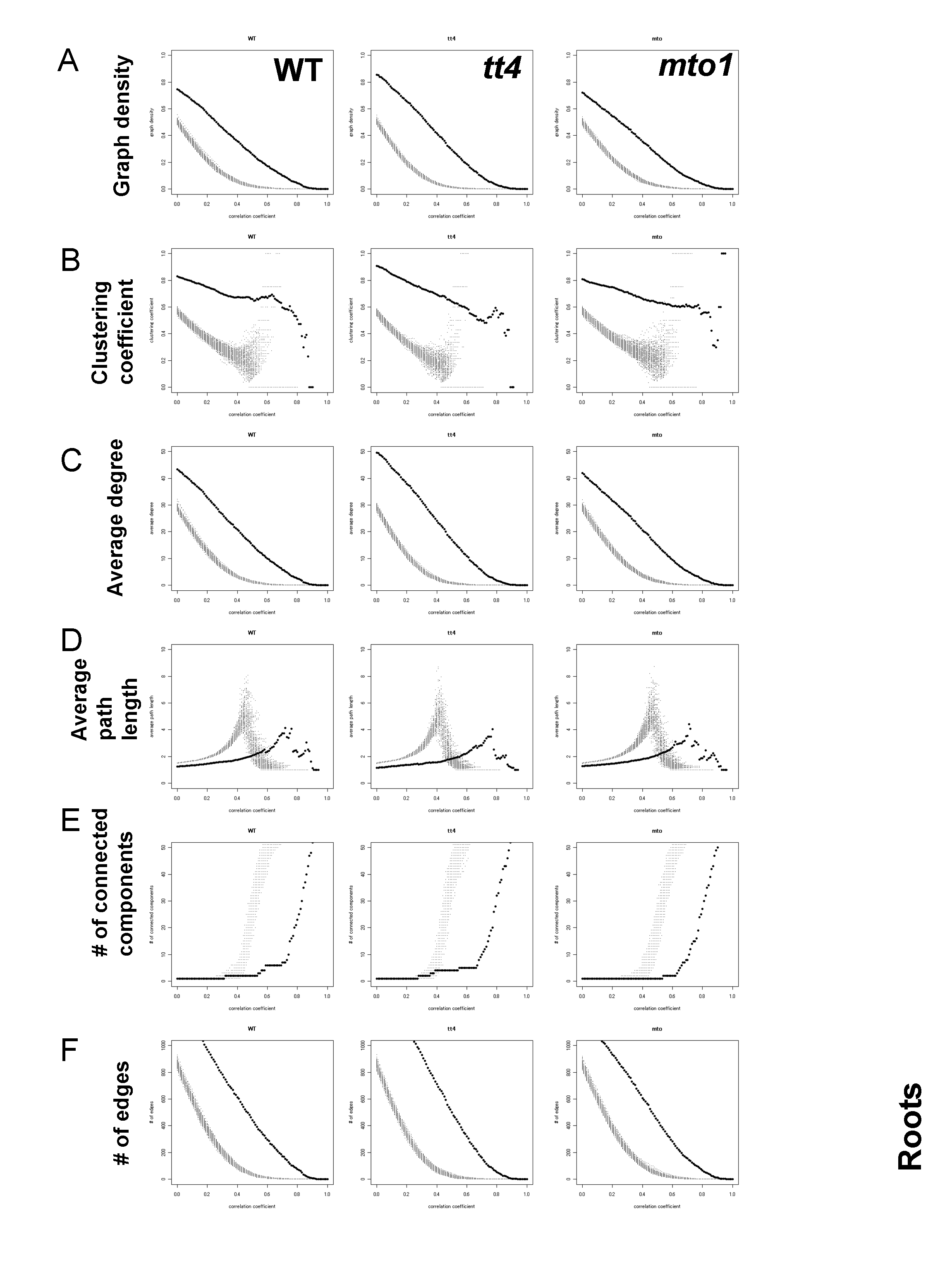

Supplement: Additional file 6 — Correlation network properties of the 59 metabolites in roots across a range of correlation coefficients. See details in the legend for Additional file 5. [file 1752-0509-5-1-S6.TIFF]
