# Supplementary material for: Metabolomic correlation-network modules in Arabidopsis based on a graph-clustering approach
Source: BMC Syst Biol. 2011 Jan 1;5:1. doi: 10.1186/1752-0509-5-1 (PMC3030539; doi:10.1186/1752-0509-5-1)
Supplement: Additional file 8 — Visualization of all DPClus clusters. See details in the legend for Figure 4. [file 1752-0509-5-1-S8.PDF]

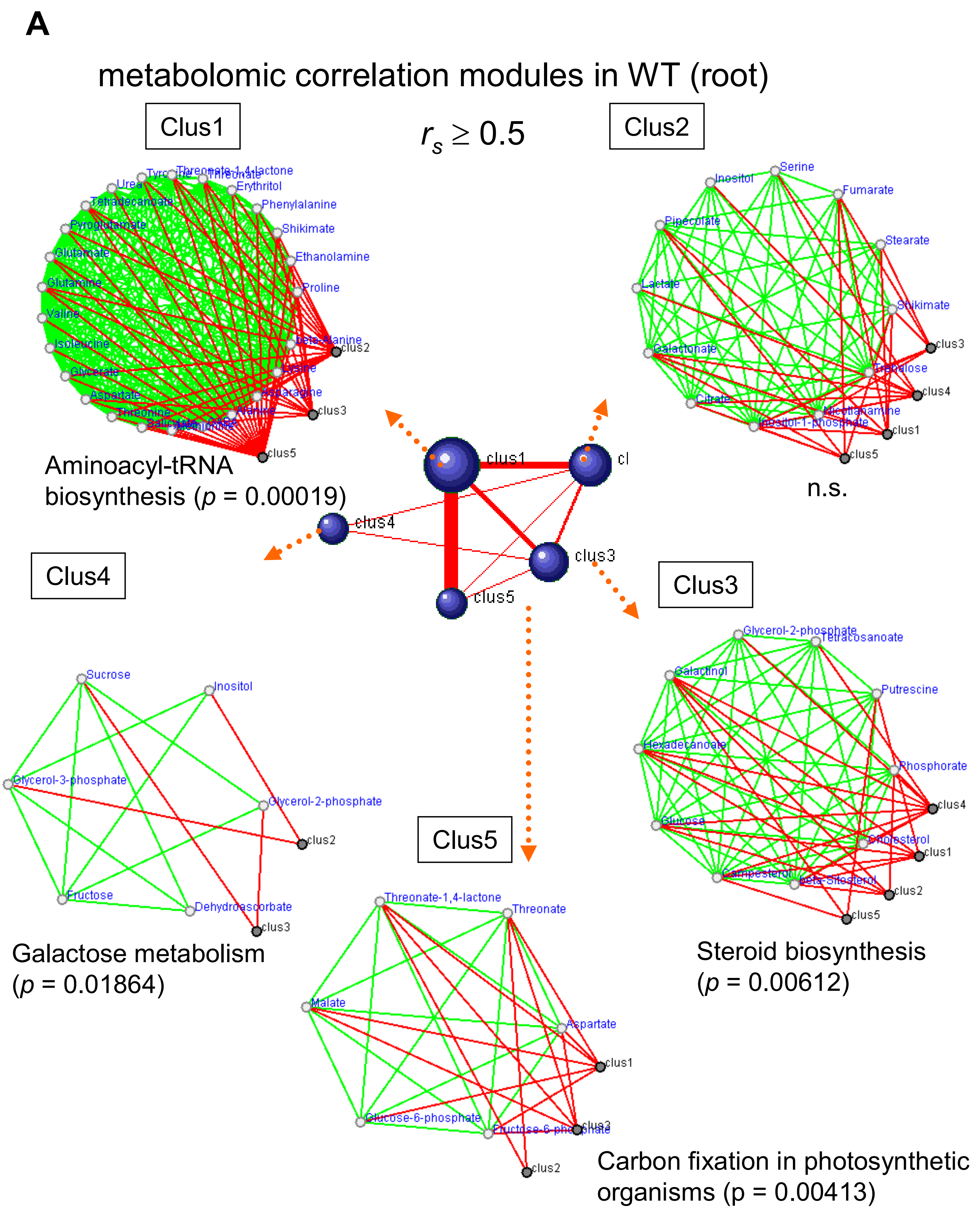
A
metabolomic correlation modules in WT (root)
$r_s \geq 0.5$
Clus1
Clus2
Clus3
Clus4
Clus5
clus1
clus3
clus4
clus5
Aminoacyl-tRNA biosynthesis (p = 0.00019)
n.s.
Galactose metabolism (p = 0.01864)
Steroid biosynthesis (p = 0.00612)
Carbon fixation in photosynthetic organisms (p = 0.00413)
Erythritol
Phenylalanine
Shikimate
Ethanolamine
Proline
Tetradecanoate
Pyroglutamate
Glutamate
Glutamine
Valine
Isoleucine
Glycerate
Aspartate
Threonine
Asparagine
Alanine
Inositol
Serine
Fumarate
Pipecolate
Stearate
Lactate
Galactonate
Trehalose
Citrate
Sucrose
Glycerol-3-phosphate
Glycerol-2-phosphate
Fructose
Dehydroascorbate
Tetracosanoate
Galactinol
Putrescine
Hexadecanoate
Phosphorate
Glucose
Cholesterol
Campesterol
beta-Sitosterol
Threonate-1,4-lactone
Threonate
Malate
Glucose-6-phosphate
clus2

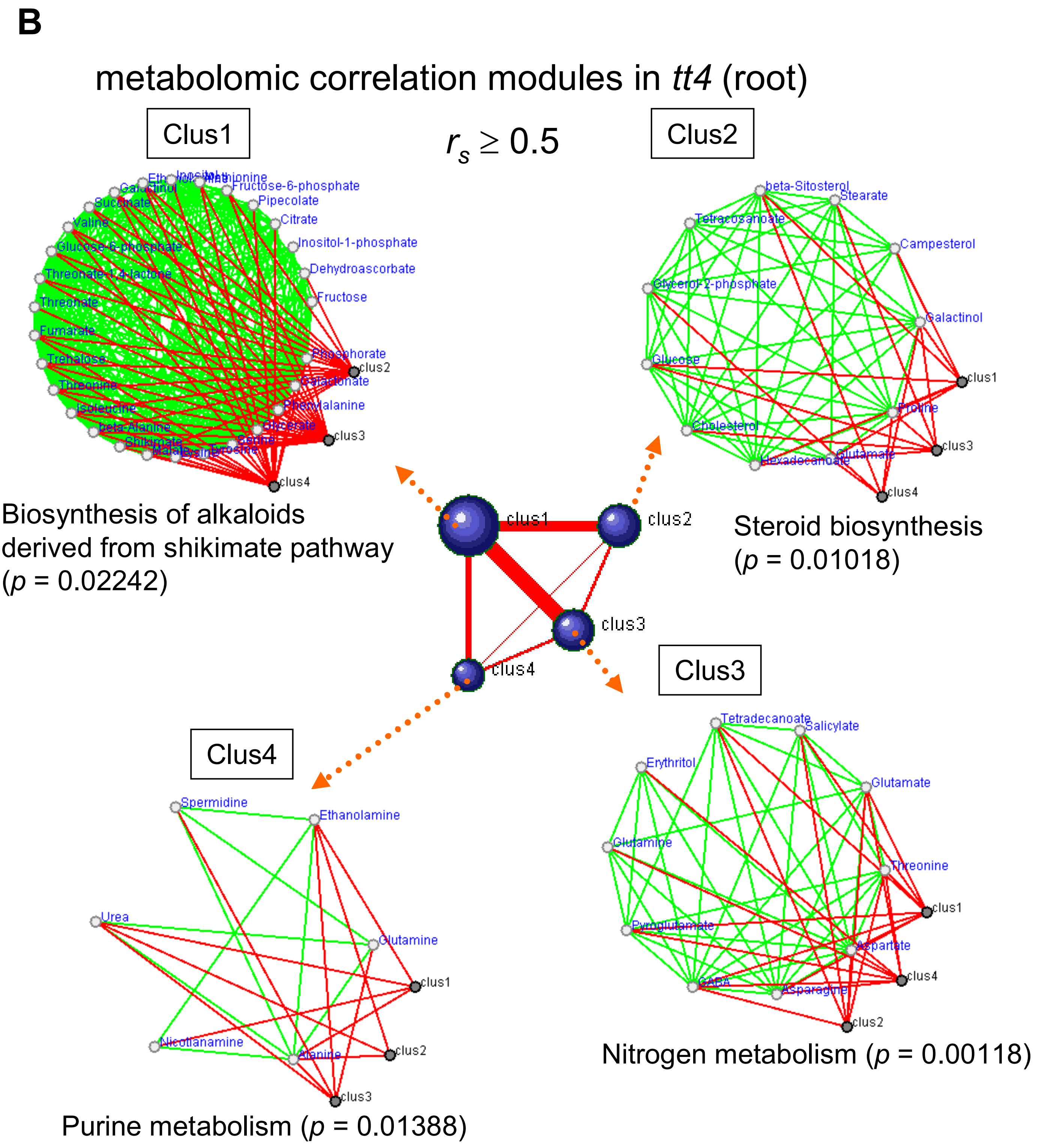

B
metabolomic correlation modules in tt4 (root)
Clus1
$r_s \geq 0.5$
Clus2
Biosynthesis of alkaloids derived from shikimate pathway (p = 0.02242)
Steroid biosynthesis (p = 0.01018)
clus1
clus2
clus3
clus4
Clus3
Clus4
Purine metabolism (p = 0.01388)
Nitrogen metabolism (p = 0.00118)
Fructose-6-phosphate
Pipecolate
Citrate
Inositol-1-phosphate
Dehydroascorbate
Fructose
Succinate
Valine
Glucose-6-phosphate
Threonate-1,4-lactone
Threonate
Fumarate
Trehalose
Threonine
Isoleucine
beta-Alanine
Phenylalanine
beta-Sitosterol
Stearate
Tetracosanoate
Campesterol
Glycerol-2-phosphate
Galactinol
Glucose
Proline
Cholesterol
Hexadecanoate
Glutamate
Spermidine
Ethanolamine
Urea
Glutamine
Nicotianamine
Alanine
Tetradecanoate
Salicylate
Erythritol
Pyroglutamate
Aspartate
GABA
Asparagine

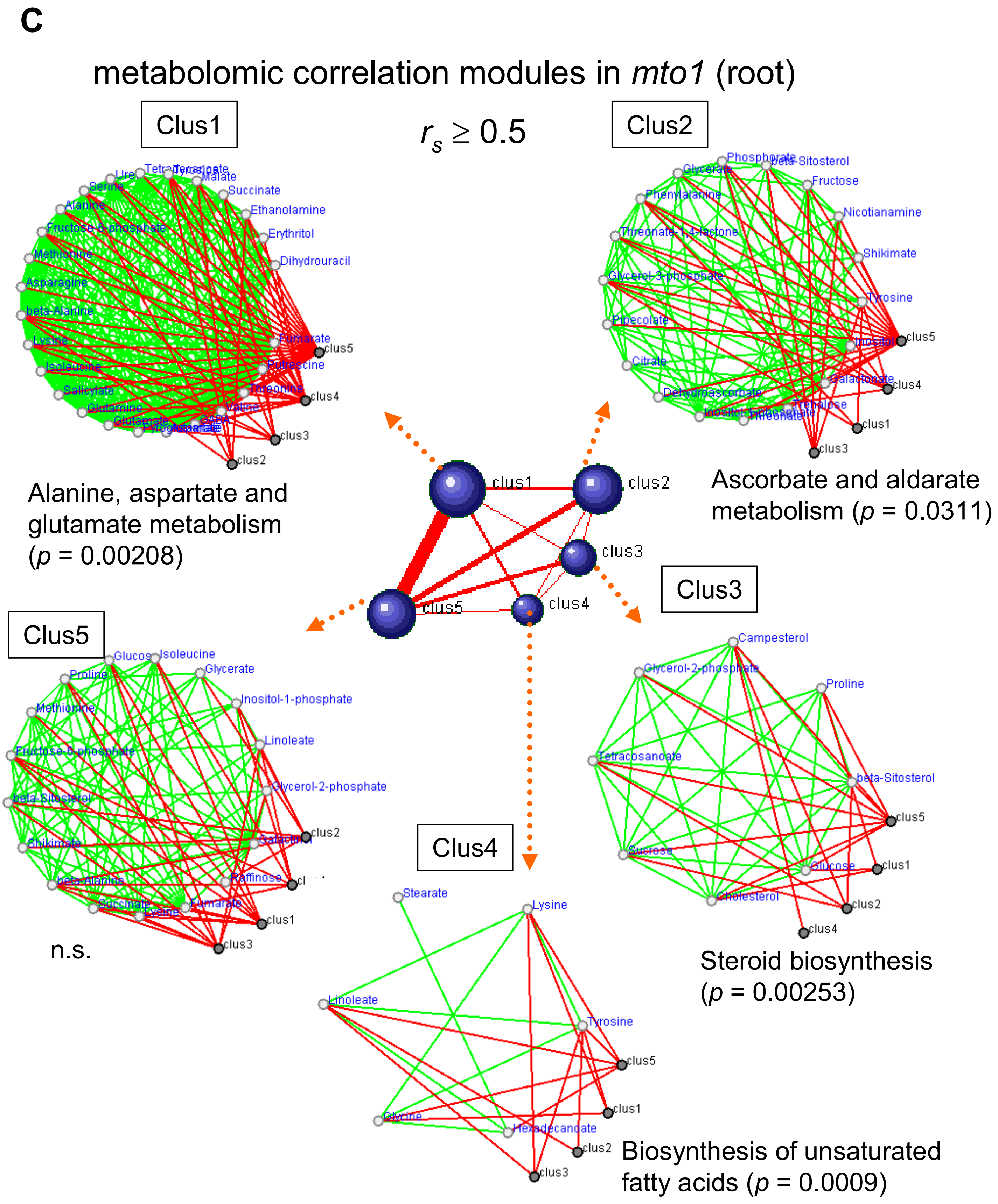
C
metabolomic correlation modules in *mto1* (root)
$r_s \geq 0.5$
Clus1
Clus2
Clus3
Clus4
Clus5
clus1
clus2
clus3
clus4
clus5
Alanine, aspartate and glutamate metabolism (p = 0.00208)
Ascorbate and aldarate metabolism (p = 0.0311)
Steroid biosynthesis (p = 0.00253)
Biosynthesis of unsaturated fatty acids (p = 0.0009)
n.s.

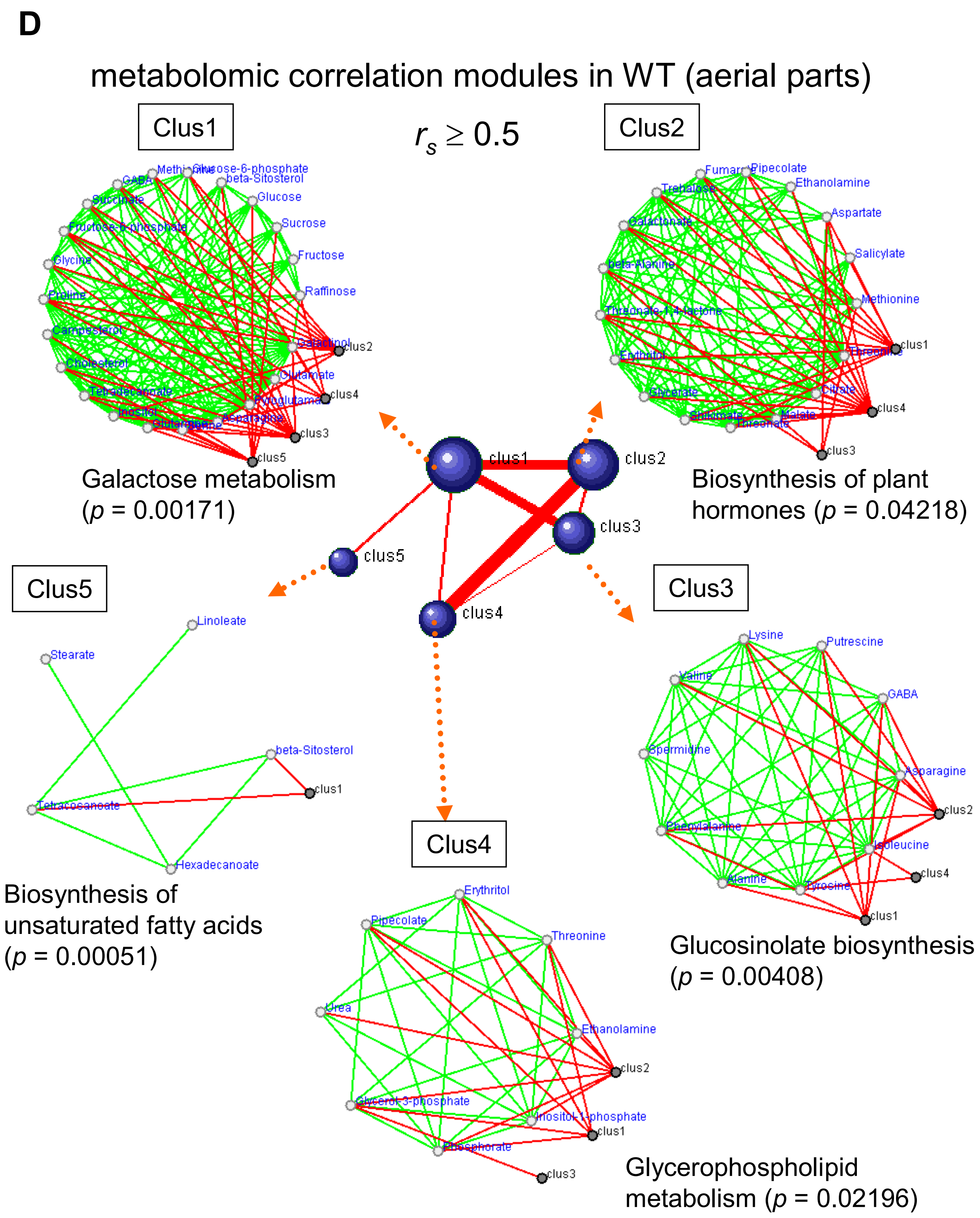
D
metabolomic correlation modules in WT (aerial parts)
$r_s \geq 0.5$
Clus1
Galactose metabolism (p = 0.00171)
Clus2
Biosynthesis of plant hormones (p = 0.04218)
Clus3
Glucosinolate biosynthesis (p = 0.00408)
Clus4
Glycerophospholipid metabolism (p = 0.02196)
Clus5
Biosynthesis of unsaturated fatty acids (p = 0.00051)
clus1
clus2
clus3
clus4
clus5
Linoleate
Stearate
beta-Sitosterol
Tetracosanoate
Hexadecanoate
Lysine
Putrescine
Valine
GABA
Spermidine
Asparagine
Phenylalanine
Isoleucine
Alanine
Tyrosine
Erythritol
Pipecolate
Threonine
Urea
Ethanolamine
Glycerol-3-phosphate
Inositol-1-phosphate
Phosphorate
Glucose-6-phosphate
Glucose
Sucrose
Fructose
Raffinose
Succinate
Fructose-6-phosphate
Glycine
Proline
Campesterol
Cholesterol
Tetradecanoate
Inositol
Glutamate
Trehalose
Ethanolamine
Aspartate
Galactonate
Salicylate
beta-Alanine
Methionine
Threonate-1,4-lactone
Erythritol
Citrate
Glycerate
Shikimate
Malate
Threonate

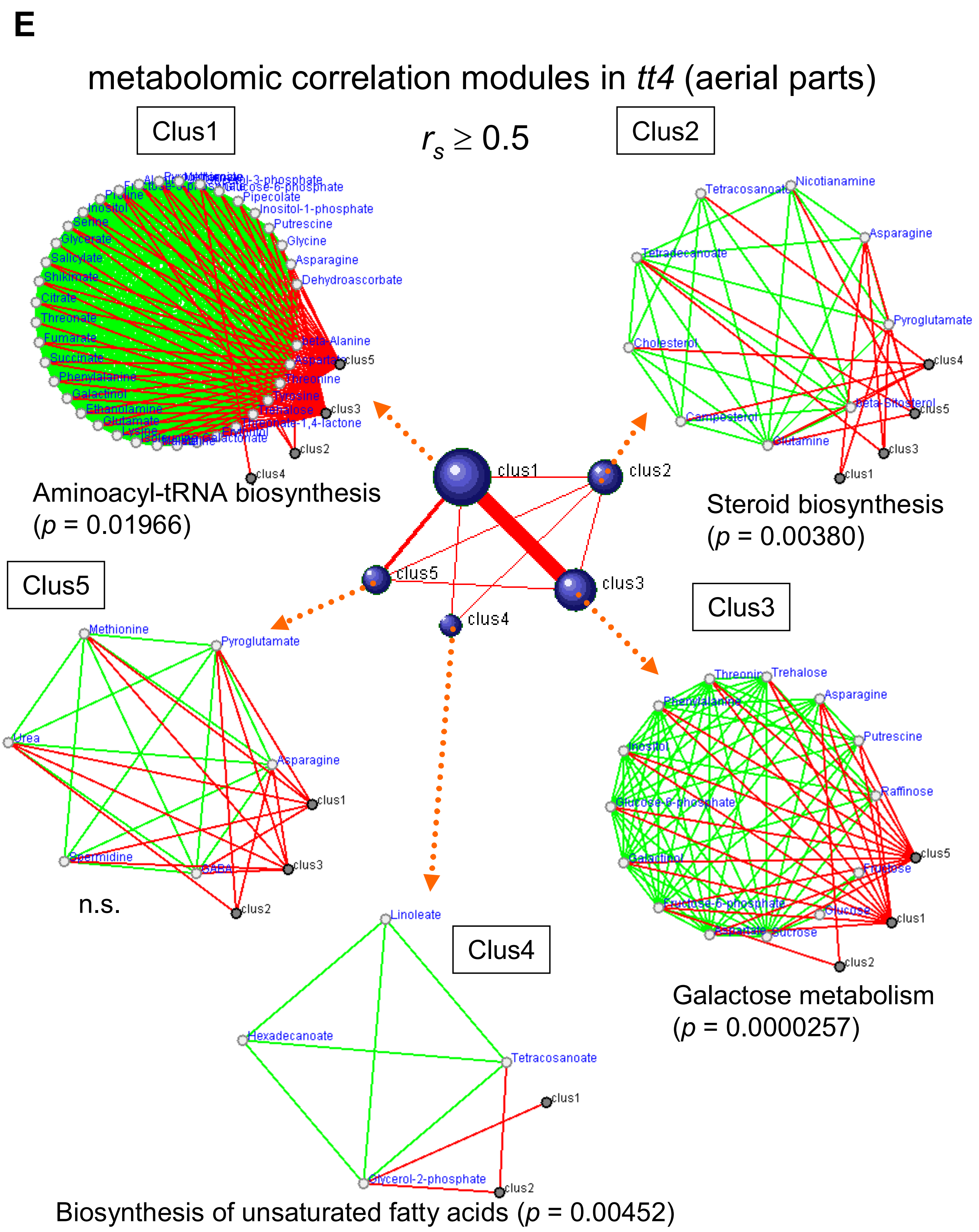
E
metabolomic correlation modules in tt4 (aerial parts)
$r_s \geq 0.5$
Clus1
Pipecolate
Inositol-1-phosphate
Putrescine
Glycine
Asparagine
Dehydroascorbate
beta-Alanine
Threonine
Tyrosine
Trehalose
Threonate-1,4-lactone
Proline
Inositol
Serine
Glycerate
Salicylate
Shikimate
Citrate
Threonate
Fumarate
Succinate
Phenylalanine
Galactinol
Ethanolamine
Glutamate
Lysine
clus5
clus3
clus2
clus4
Aminoacyl-tRNA biosynthesis
(p = 0.01966)
Clus2
Tetracosanoate
Nicotianamine
Asparagine
Tetradecanoate
Pyroglutamate
Cholesterol
clus4
beta-Sitosterol
clus5
Campesterol
Glutamine
clus3
clus1
Steroid biosynthesis
(p = 0.00380)
clus1
clus2
clus5
clus3
clus4
Clus5
Methionine
Pyroglutamate
Urea
Asparagine
clus1
Spermidine
GABA
clus3
clus2
n.s.
Clus3
Threonine
Trehalose
Phenylalanine
Asparagine
Putrescine
Inositol
Raffinose
Glucose-6-phosphate
clus5
Galactinol
Fructose
Fructose-6-phosphate
Glucose
clus1
Aspartate
Sucrose
clus2
Galactose metabolism
(p = 0.0000257)
Clus4
Linoleate
Hexadecanoate
Tetracosanoate
clus1
Glycerol-2-phosphate
clus2
Biosynthesis of unsaturated fatty acids (p = 0.00452)

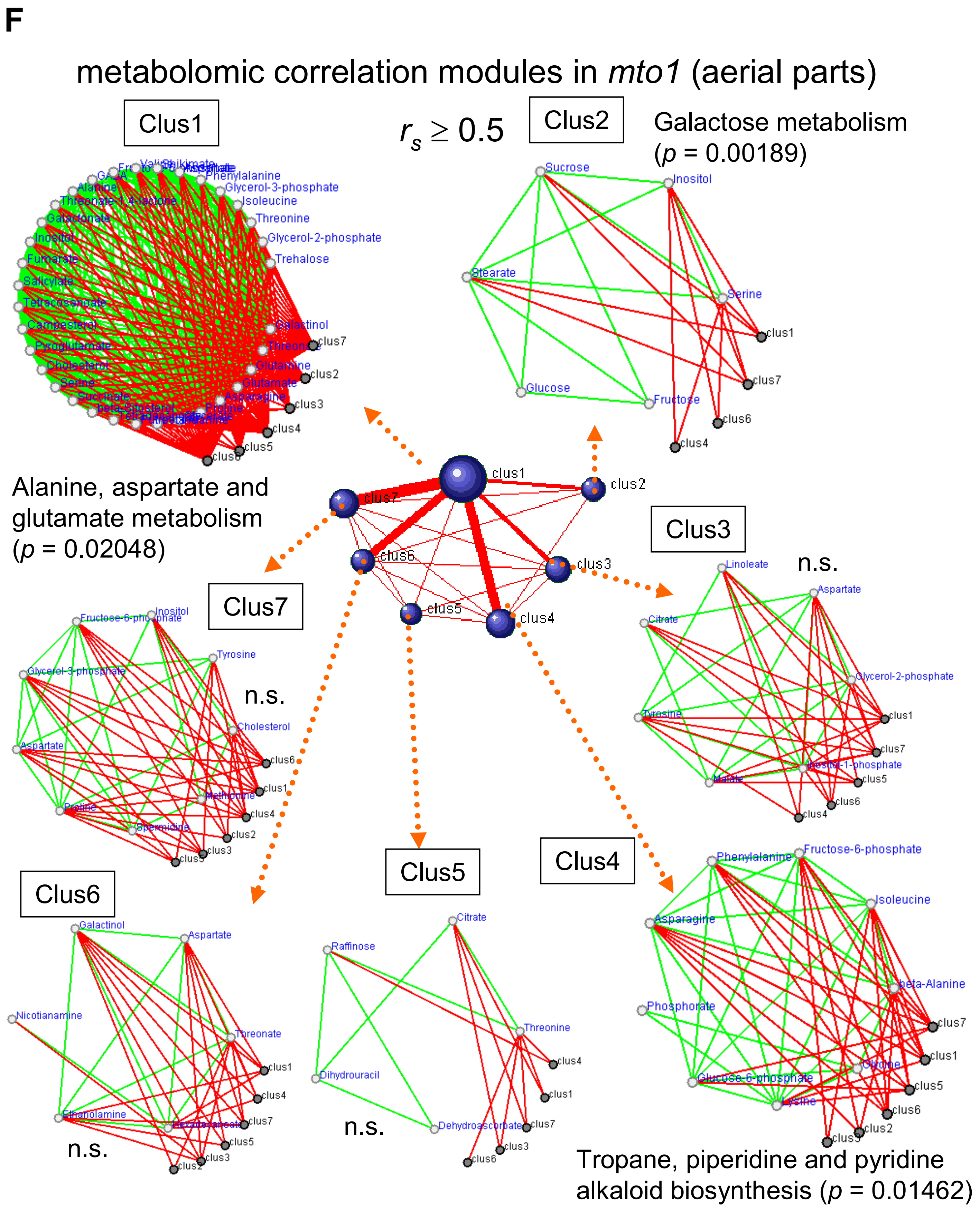
F
metabolomic correlation modules in mto1 (aerial parts)
$r_s \geq 0.5$
Clus1
Clus2
Galactose metabolism
(p = 0.00189)
Sucrose
Inositol
Stearate
Serine
Glucose
Fructose
Alanine, aspartate and
glutamate metabolism
(p = 0.02048)
Clus3
n.s.
Linoleate
Aspartate
Citrate
Glycerol-2-phosphate
Tyrosine
Inositol-1-phosphate
Malate
Clus7
n.s.
Fructose-6-phosphate
Inositol
Glycerol-3-phosphate
Tyrosine
Cholesterol
Aspartate
Methionine
Proline
Spermidine
Clus6
Galactinol
Aspartate
Nicotianamine
Threonate
Ethanolamine
n.s.
Clus5
Citrate
Raffinose
Threonine
Dihydrouracil
Dehydroascorbate
n.s.
Clus4
Phenylalanine
Fructose-6-phosphate
Isoleucine
Asparagine
beta-Alanine
Phosphorate
Glycine
Glucose-6-phosphate
Lysine
Tropane, piperidine and pyridine
alkaloid biosynthesis (p = 0.01462)
Phenylalanine
Glycerol-3-phosphate
Isoleucine
Threonine
Glycerol-2-phosphate
Trehalose
Galactinol
Glutamine
Glutamate
Asparagine
Proline
Alanine
Galactonate
Inositol
Fumarate
Salicylate
Campesterol
Pyroglutamate
Cholesterol
Serine
Succinate
clus1
clus2
clus3
clus4
clus5
clus6
clus7
